# Graceful Self-Promotion: The impact of a short faculty development session

**DOI:** 10.15694/mep.2020.000024.1

**Published:** 2020-02-06

**Authors:** Katherine Huber, John Huber, Zareen Zaidi

**Affiliations:** 1 University of Florida; 2 University of Notre Dame

**Keywords:** Graceful Self-Promotion, Faculty Development Workshop, Imposter Syndrome

## Abstract

This article was migrated. The article was marked as recommended.

Self-promotion can be challenging for physicians who are looking to advance their careers. While they want to make their successes in the workplace known, they are afraid of coming off as aggressive and turning off the people that they are trying to impress with their accomplishments. This dilemma led to the coining of the term “graceful self-promotion” (GSP), a method of making one’s accomplishments and abilities visible with tact and humility. The Division of General Internal Medicine at the University of Florida undertook a faculty development session focusing on GSP skills. The session started with participants interacting with each other using Bingo cards which listed GSP strategies in order to facilitate discussion. This was followed by an interactive discussion on barriers to career advancement and strategies to practice GSP. Changes in physician knowledge regarding self-promotion techniques and attitudes towards its importance were assessed using statistical tests from responses to pre- and post-surveys. We measured a positive change in physician attitudes towards their ability to self-promote following the information session (p-value < 0.005). Perceptions about the importance of self-promotion activities improved as well (p-value < 0.005). Participants comments revealed greater understanding of need for networking, developing a spirit of generativity, and having a prepared Elevator Speech. In this era where pressure to generate clinical revenue allows for limited faculty development time even a short 1-hour session can create awareness about importance of GSP for academic advancement, strategies for participants to use, and awareness of barriers to GSP.

## Introduction

Physicians in academic medicine face many challenges in advancing their careers in light of the multiple demands on their time, including clinical work, educational responsibilities, and research. Many are not aware of self-promotion techniques and their importance in career development. They are reticent to make their successes known, as they fear being viewed as aggressive and having the opposite effect on the people that they are trying to impress with their accomplishments. However, achievements and positive attributes, when presented at the appropriate times and during appropriate situations, can help with career advancement. Nevertheless, a less-than-positive result can occur, particularly for women and minorities, if achievements are presented in an exaggerated and over exuberant manner or too frequently ( Rudman, 1998). Unfortunately, studies have shown that those who practice more aggressive self-promotion fail to realize that the negative effects outweigh the positive on the recipient ( Scopelliti, Loewenstein and Vosgerau, 2015). As a result, faculty need to ensure that they present their successes and achievements with humility and in a way that is not perceived to be irritating or boastful in order to get the recognition that they deserve. The term “graceful self-promotion” (GSP) was developed in response to this dilemma as a method of making visible one’s accomplishments and abilities with tact and humility ( Page Morahan, 2004).

The need for physicians to learn to self-promote is important for several reasons. Due to long-standing cultural and gender roles, women and minorities tend to have difficulty utilizing the techniques of GSP. Additionally, physicians often feel that if they work very hard and focus their efforts in their particular area of expertise, their performance will be noticed and they will be given opportunities for advancement within their division, department, or college. This approach of not making one’s accomplishments public, unfortunately, is generally not successful and leads to frustration when they are not chosen for leadership roles or other positions important in advancing their careers.

In an effort to create awareness about the concept of GSP among faculty in the Division of General Internal Medicine (GIM) at the University of Florida College of Medicine, we designed a one-hour interactive faculty development workshop on GSP. The specific aim of the study was to evaluate the impact of a single short workshop on faculty’s understanding and perceived benefit of GSP.

## Methods

### Setting and participants

The University of Florida is located in Gainesville, Florida. The teaching hospital serves as a tertiary care center for several surrounding counties as well as the rest of the state. The Division of General Internal Medicine consisted of 40 faculty members in 2018. Of these faculty members, there were 27 assistant professors, 8 associate professors, and 5 full professors. Additionally, the Division included two physician assistants. As the hospital has expanded significantly between 2016-2020, 12 new faculty members (most of which are early-career) have been hired in the Division. In 2018, 23 faculty members in the Division were women.

Given the demographics of the Division, we sought to create awareness among faculty about issues that impede academic growth and to discuss GSP strategies that may address these issues. Twenty-four faculty members attended the GSP faculty development session. Demographic data regarding gender, race, age and years of experience of the attendees were collected and are provided in Table 1. The objectives of the workshop were to help faculty identify barriers that potentially hinder career advancement and develop strategies to practice graceful self-promotion.

**Table 1.** Demographic Data of GSP Workshop Participants.

Category		Count (n)
Gender	Male	7
	Female	14
Race	Asian	6
	White	14
	Other	1
Age	25-35	7
	36-45	8
	46-55	4
	56-65	2
Years of Experience	0-5	8
	6-10	4
	11-15	2
	16-20	4
	21-25	1
	> 25	2

The gender, race, age, and years of work experience are reported for the GSP Workshop participants conducted in the Division of General Internal Medicine at the University of Florida.

**Figure 1.**
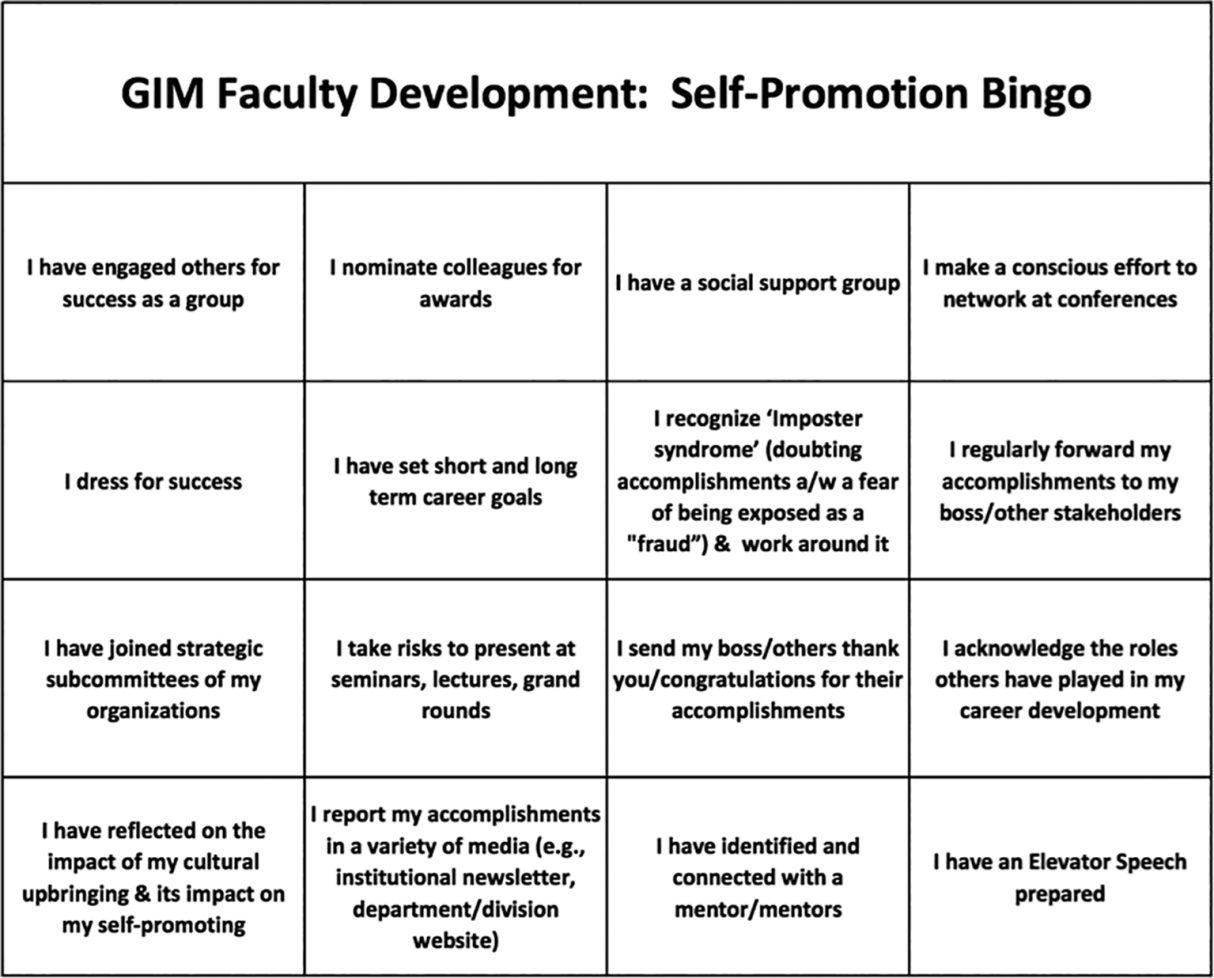
Example of a GSP Bingo Card. A GSP Bingo Card used in the GSP Workshop is shown.

The one-hour session began with participants walking around the room interacting with other participants using “Bingo” cards, which listed GSP strategies ( Figure 1). The objective of this exercise was for the participants to discover if other participants had previously used these strategies and, if so, to share experiences. As in the game “Bingo”, the first participant to check all boxes in a row horizontally, vertically or across was asked to call out “Bingo.” A de-briefing in the form of an interactive presentation and discussion followed on the barriers that hinder career advancement and appropriate strategies to practice GSP.

Faculty members were asked to discuss GSP with others and check a box if someone had previously practiced one of the strategies mentioned in the box.

### Data Collection and Analysis

The study was approved by the University of Florida Institutional Review Board (IRB201702485).

An IRB-approved anonymous survey was distributed to all participants before the session inquiring about how equipped they were to practice GSP, their understanding of GSP, any previous techniques that they may have used, and how important they felt GSP is for career advancement ( Table 2). A Likert scale of 1 to 5 with ‘1’ being not important at all and ‘5’ being very important was used ( Albaum, 1997). A post-session survey asked participants how likely they were to use GSP techniques, which techniques they would use, and their change in perception concerning the importance of GSP. Space for free responses was also provided. Simple statistics were used to analyze the demographic data, and a paired t-test was used to assess changes in physician knowledge regarding self-promotion techniques and attitudes towards GSP. An inductive qualitative analysis was used to analyze the open-ended comments.

**Table 2.** Survey questions asked during GSP Workshop.

Before Intervention
What do you understand about self-promotion in academics?
What techniques have you used to advance your career?
How important do you think self-promotion is in advancing your career?
**After Intervention**
What do you understand about self-promotion in academics after hearing this talk?
How likely are you to use the techniques presented moving forward?
Out of the techniques described, which would you feel comfortable using?
After hearing the talk, how important do you think self-promotion is in advancing your career?

Pre- and post-workshop survey questions are provided. Where appropriate, responses were in the form of a Likert Scale of 1 to 5 in which a response of 1 signified “not important at all” and a response of 5 signified “very important.”

## Results

Twenty-four faculty members attended the session with 20 faculty members completing the survey (response rate: 83%). Of all participants, 66% identified as female. Moreover, 62% were white, 29% were of Asian descent, and 9% declared as other ( Table 1). Seventy-one percent of the participants were between the ages of 25-45. Sixty-seven percent of the participants had between 0-15 years of experience and 15% had over 20 years of experience. By academic position, 68% percent of the participants were assistant professors, 21% were associate professors and 11% were physician assistants.

There was a positive change in physician attitude regarding their perceived ability to self-promote (p < 0.005) ( Table 3). Participants also had an increased understanding about the importance of self-promotion (p < 0.005). We measured statistically significant, positive changes for female faculty with regards to understanding the importance of GSP and perceived ability to self-promote (p < 0.005). A positive change was measured for male faculty but was not statistically significant. This is likely due to the small sample size for male faculty.

**Table 3.** Statistical analysis of GSP workshop.

Description		Sample Size (n)	Test Statistic (t)	p-value
Importance of GSP	Overall	20	3.94	**< 0.005**
	Male	6	1.58	0.087
	Female	14	3.68	**< 0.005**
Equipped with strategies for GSP	Overall	19	6.88	**< 0.005**
	Male	6	2.00	0.051
	Female	13	8.83	**< 0.005**

Results and sample sizes of pair-wise t-tests are reported for changes in physicians’ attitudes towards the importance of GSP and their knowledge of strategies to practice GSP. Statistical tests were performed and stratified by gender. Statistical significance was assessed at 0.05 significance, and statistically significant results are bolded.

A thematic analysis of the comments revealed two main themes “Increased Awareness of GSP” and “Planned Changes” ( Table 4). Participants commented about how the workshop helped them become aware of the concept of GSP:

“Self-promotion is an important tool. You cannot expect to have things handed to you. You need to make your accomplishments know in a graceful way” P01

They noted an increased understanding of the need for networking, creating a Power Map ( Clark, 2012), developing a spirit of generativity, and having a prepared “Elevator Speech” to highlight their accomplishments ( Page Morahan, 2004). Additionally, participants noted that they were now aware of the Imposter Syndrome and would actively use strategies to combat it ( Fitzpatrick and Curran, 2014). Participants noted several changes they would make for career advancement, including “dressing for success”, seeking out opportunities for career advancement, and collaborating with others. For example, one participant planned to:

“Seek opportunities that make me uncomfortable in an effort to overcome a strong sense of the Imposter Syndrome” P07

**Table 4.** Thematic analysis of comments.

Themes	Representative Quotes
Increased awareness	“Self-promotion is an important tool. You cannot expect to have things handed to you. You need to make your accomplishments know in a graceful way.”
	“It is important to assure that I am being recognized for what I am doing and to search out new challenges and opportunities.”
	“Self-promotion is to be self-aware, proactive and to promote oneself.”
	“I had not thought about this before but praising others and congratulating them on their awards and accomplishments can help lead to a collaborative environment in which we all promote each other.”
	“It is important to assure that I am being recognized for what I am doing and to search out new challenges and opportunities.”
Changes planned	“I understand so much more about Imposter Syndrome- I will try to be aware of this and combat it.”
	“Seek opportunities that make me uncomfortable in an effort to overcome a strong sense of the Imposter Syndrome.”
	“Collaboration with our colleagues in research projects. I will try to improve my ‘dress for success.’ Be a role model and mentor others.”
	“Self-promotion will take preparedness, but gradual accumulation of mentors and colleagues will be the first step for me (Power Map).”
	“Really liked the focus on promoting, supporting others. Will do more of that.”

Themes addressed in the post-intervention survey are presented along with representative quotes highlighting these themes.

## Discussion

There are three main findings from this study. First, a short faculty development workshop can create awareness, in this case, about GSP and issues that impede faculty advancement. Second, such workshops can equip faculty with tools for GSP. Third, women faculty members appear to benefit more from GSP training than their male colleagues though this difference may be due only to a small sample size.

Despite evidence in the literature supporting GSP as a very effective tool for career advancement, it is underutilized. Learning the art of GSP is essential at the clinical, academic, administrative, and societal level ( Bleier and Kann, 2013). The number of women in the workforce has significantly increased in the past few decades, yet the Committee on Maximizing the Potential of Women in Academic Science and Engineering found that female faculty members receive lower compensation, experience lower rates of promotion, and hold fewer leadership roles than their male colleagues ( Arora, 2016). Additionally, even after adjusting for specialty, academic rank, leadership position, and research time, male gender is associated with a higher salary with male physician researchers receiving $13,000 more on average ( Jagsi *et al.*, 2012). Drivers of this phenomenon may include for women a tendency to underestimate their skills, reluctance to self-promote or to negotiate one’s salary, and the potential to suffer from the “Imposter Syndrome” ( Ross-Smith and Chesterman, 2009). More women than men suffer from “Imposter Syndrome”, which involves the perception of themselves as less capable and associated with a feeling of self-doubt and a constant fear of being exposed “as a fraud” ( LaDonna, Ginsburg and Watling, 2018).

In this study conducted in the Division of General Internal Medicine at the University of Florida, a majority of the participants were younger women and junior faculty. However, the study did include males and more senior faculty. The study participants were culturally diverse with 34% of Asian or other descent. While we found that younger faculty members and assistant professors were less informed about GSP, we did see a significant improvement in the knowledge of the knowledge of GSP and likelihood of using the techniques after the intervention, particularly among women.

The study’s limitations include its small sample size and an overrepresentation of women and young faculty at the start of their career. While the overrepresentation of this demographic among study participants may limit the generalization of this findings to other demographics, it is also important to note that this is the population who is most likely to benefit from such training. Additionally, we did not ask if the participants had attended any type of intervention, faculty development session or conference in the past, which could have impacted the responses. We did not measure long-term impact of the intervention because the purpose of the workshop was to create awareness of GSP. Follow-up sessions and workshops offered to women and minorities are being developed by the Women in Medicine group at the University of Florida. Studying the impact of these follow-up sessions and workshops would be an area for further research.

## Conclusions

Despite proven benefits of GSP, few academic general internists in our Division felt knowledgeable about techniques involved as well as the importance of this strategy. In this current era of academic medicine where pressure to generate clinical revenue allows for limited faculty development time, even a short 1-hour session can create awareness about the importance of GSP for academic advancement strategies for participants to use and raise awareness of barriers to GSP. Follow up in depth sessions allowing hands-on practice to reinforce methods for GSP on a regular basis may be useful.

## Take Home Messages

• Graceful self-promotion is an important tool for advancement in academic medicine that is under-recognized and under-utilized by faculty.
• A short interactive faculty development session can increase faculty understanding of the need for and benefits of self-promotion and equip them with strategies to utilize in their workplace.
• Follow-up sessions with more in-depth education on self-promotion techniques may be of benefit in reinforcing the specific techniques and encouraging their utilization by faculty.

## Notes On Contributors

Dr. Katherine Huber is an Assistant Professor in the Division of General Internal Medicine at the University of Florida.

Mr. John Huber is a PhD student at the University of Notre Dame.

Dr. Zareen Zaidi is a Professor in the Division of General Internal Medicine at the University of Florida.

## Declarations

The author has declared that there are no conflicts of interest.

## Ethics Statement

The study was approved by the University of Florida Institutional Review Board (IRB201702485) on March 27th, 2019.

## External Funding

This article has not had any External Funding
