# Supplementary material for: Graceful Self-Promotion: The impact of a short faculty development session
Source: MedEdPublish (2016). 2020 Feb 6;9:24. [Version 1] doi: 10.15694/mep.2020.000024.1 (PMC10697563; doi:10.15694/mep.2020.000024.1)
Supplement: Peer review history

# Reviewer report: Reviewer response for version 1

**DOI:** 10.21956/mep.19984.r30403

**Published:** 2020-03-06

**Authors:** P Ravi Shankar

**Affiliations:** 1 American International Medical University

This review has been migrated. The reviewer awarded 4 stars out of 5 This is an interesting manuscript addressing the issue of graceful self-promotion (GSP). Though I was broadly aware of the concept the term GSP is a new one for me. The study is well-written and the Methodology has been described in detail. I would be interested in knowing more details about the interactive faculty development session. Some information has been provided in the paper but it will be nice if the authors can provide a table with the details of the session. Some of the terms like ‘power map’, ‘spirit of generativity’ may need greater explanation. This article would be of interest to a wide range of medical educators.

Reviewer Expertise:

NA

No decision status is available

# Reviewer report: Reviewer response for version 1

**DOI:** 10.21956/mep.19984.r30402

**Published:** 2020-02-21

**Authors:** Trudie Roberts

**Affiliations:** 1 Leeds Institute of Medical Education

This review has been migrated. The reviewer awarded 5 stars out of 5 I very much enjoyed reading this paper. It covers a very important topic. Throughout a long career in academia I have seen good thoughtful colleagues both men and women passed over in favour of those less able but with an ability to push themselves forward. So helping individuals to be able to get recognition for their work and expertise is of interest to me and should be to organisations.I would have liked to know more about the content of the workshop and perhaps the authors could do a follow up containing an in depth practical workshop plan

Reviewer Expertise:

NA

No decision status is available
